# Toll-like receptor 2 impacts small intestinal villus capillarization through epithelial dual oxidase 2

**DOI:** 10.1016/j.redox.2026.104212

**Published:** 2026-05-14

**Authors:** Nadja Paeslack, Maximilian Mimmler, Jana Schulz, Julia Kownatzki, Bettina Kollar, Florentina Melzow, Julie Zamor, Olga Dremova, Marin Kuntic, Klytaimnistra Kiouptsi, Natalia Soshnikova, Amrit Mann, Jens M. Kittner, Andreas Daiber, Felix Sommer, Christoph Reinhardt

**Affiliations:** a Center for Thrombosis and Hemostasis (CTH), University Medical Center, Johannes Gutenberg-University Mainz, Mainz, Germany; b German Center for Cardiovascular Research (DZHK), Partner Site Rhine-Main, Mainz, Germany; c Department of Operative, Preventive and Pediatric Dentistry, University Medicine Berlin, Charité, Berlin, Germany; d Department of Cardiology, University Medical Center, Johannes Gutenberg University Mainz, Mainz, Germany; e Research Center for Immunotherapy (FZI), University Medical Center of the Johannes Gutenberg-University Mainz, Germany; f Helios Klinikum Erfurt, Erfurt, Germany; g Institute of Experimental Medicine (IEM), Christian-Albrechts-University, Kiel, Germany

**Keywords:** Toll-like receptor-2, Dual oxidase 2, Inflammatory bowel disease, Capillaries small intestine, Microbiota

## Abstract

The microbiota shapes postnatal gut development and physiology. In the small intestine, epithelial-to-endothelial crosstalk governs the microbiota-induced remodeling of villus capillary networks essential for nutrient transport. The intestinal epithelial enzyme dual oxidase 2 (DUOX2), an established regulator of the microbiome-host interaction, exerts microbicidal functions through the generation of reactive oxygen species. However, its role in intestinal vascular development remains poorly understood. Here, we demonstrate a Toll-like receptor 2 (TLR2)-dependent regulatory pathway controlling DUOX2 expression that influences villus vascularization in the small intestine. Mice globally lacking DUOX2 activity exhibited a notable reduction in vascularization in the small intestine, accompanied by alterations in gut microbial community structure. Conversely, mice with an intestinal epithelial-specific deficiency of TLR2 displayed an increase in villus vascularization along with elevated expression levels of DUOX2. Notably, DUOX2 expression was strongly upregulated in intestinal epithelial biopsies from patients with Crohn's disease. Similarly, inflammatory conditions induced by dextran sulfate sodium (DSS) treatment in mice resulted in increased epithelial *Duox2* expression accompanied by enhanced villus vascularization. Together, our findings suggest a microbiota–TLR2–DUOX2 signaling axis in intestinal epithelial cells that promotes villus vascularization. This mechanism links microbial sensing in the intestinal epithelium to structural remodeling of the villus microvasculature during homeostasis and inflammation.

## Highlights

• Epithelial TLR2 regulates DUOX2 expression and mucosal vascularization.
• Intestinal epithelial *Duox2*-deficiency is linked to reduced blood capillaries in the small intestinal mucosa.
• Acute intestinal inflammation is associated with increased *Duox2* expression and small intestinal villus vascularization.

## Graphical abstract

**Figure:**
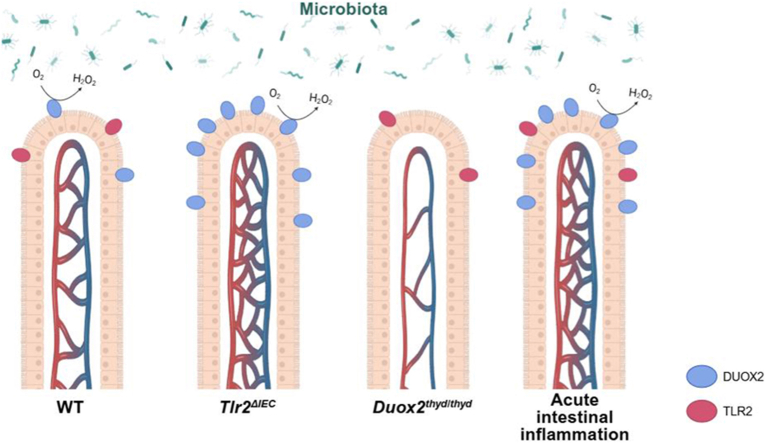

## 1 Introduction

The commensal gut microbiota is established at birth, representing one of the most densely colonized microbial ecosystems [1] [2]. In addition to its broad impact on host physiology [3], this gut resident ecosystem has been described to influence the development of many inflammatory disease states, including inflammatory bowel disease (IBD) [4] [5]. Crohn's disease patients are characterized by a dysbiotic gut microbiota [6] [7]. Chemically induced colitis reduces the diversity of the gut resident microbiota and increases the relative abundance of the *Enterobacteriaceae* [8]. Intestinal inflammation elicited by a colitogenic microbiota is a transmissible trait, as demonstrated by colitis-predisposed genetic mouse models [9] [10]. Therefore, fecal microbiota transplantation is currently explored in clinical studies for the treatment of IBD [11].

Many of the microbiota-induced effects on intestinal physiology are triggered by Toll-like receptors (TLRs) of the intestinal epithelium [12], which are pivotal regulatory elements for the immune balance in intestinal immune homeostasis and repair processes [13] [14]. Remodeling processes in IBD are triggered by innate immune pathways [13]. In particular, the microbiota-induced upregulation of TLR2 has been shown to augment renewal of the small intestine [15] [16]. Interestingly, inflammation-induced angiogenesis in the intestinal mucosa is a recognized feature in IBD [17] [18]. In the small intestine, the microbiota significantly influences the remodeling of villus capillary networks [19] [20] [21], which are essential for dietary-derived nutrient transport, through epithelial-to-endothelial crosstalk [22] [23]. TLRs are critically involved in the control of intestinal angiogenesis under physiological and inflammatory conditions, but so far it is unresolved how innate immune sensing is integrated into angiogenic signaling cues in the gut mucosa [24] [25]. Interestingly, in mouse models of chemically induced acute colitis [26], mucosal angiogenesis is augmented by the increased expression of angiogenic factors such as vascular-endothelial growth factor (VEGF) and placental growth factor (PLGF), which have a protective role and determine the severity of the inflammatory phenotype [27]. Furthermore, in the dextran sulfate sodium (DSS)-induced mouse model it has been demonstrated that the deficiency of TLR2 or MyD88 in the intestinal epithelium markedly reduces regeneration and spontaneous tumor development in the colon [16]. Superoxide production by NADPH oxidases, which are upregulated by TLR signaling [28], contributes to the onset of IBD [29]. However, the involved signaling mechanisms of how sensing of gut microbiota through pattern recognition receptors affects remodeling of intricate capillary networks in the small intestinal mucosa in the setting of intestinal inflammation remain ill defined.

A crucial enzyme responsible for hydrogen peroxide (H_2_O_2_) production on the gut epithelial surface is the NADPH-oxidase family member Dual Oxidase 2 (DUOX2) [30] [31]. DUOX2 is a key epithelial enzyme generating superoxide as an intermediate product through electron transfer from NADPH to oxygen, which is rapidly converted into H_2_O_2_ on the luminal site through its N-terminal peroxidase-homologue domain. Interestingly, DUOX2 was uncovered as one of the strongest upregulated genes in the colonic mucosa of Crohn's disease patients [32] [33] [34]. Germ-free (GF) mouse models consistently revealed a gut microbiota dependent upregulation of this epithelial-expressed hydrogen peroxide producing oxidase [35] [36]. While *Trif*-deficiency and intestinal epithelial deficiency of the NF-κB subunit p65 showed reduced DUOX2 expression in the ileum, *Myd88*-deficiency did not result in reduced expression of the oxidase [35]. A significantly increased induction of DUOX2 was observed when GF mice were associated with a dysbiotic gut microbiota from patients with active colitis compared to microbiota from healthy donors [37]. Furthermore, the changes in gene expression in *DUOXA2*-deficient mice resembled those in non-inflamed ilea of IBD patients [37]. To date, the interplay of the gut microbiota and Toll-like receptors (TLRs) in the regulation of gut epithelial DUOX2 expression and its implications in intestinal morphogenesis and repair remains elusive.

Here, we provide first insights on how epithelial TLR2, by regulating the expression of DUOX2, impacts villus vascularization in the small intestine. *Duox2*-deficiency was associated with a changed microbiome in the small intestinal fecal content. In our analyses of clinical specimens of the inflamed terminal ileum of Crohn's patients, we found that increased DUOX2 expression is associated with acute inflammation. Understanding these complex interactions offers new insights into the intricate mechanisms behind gut morphogenesis and vascular network regulation.

## 2 Material and methods

### 2.1 Mice

Mice lacking TLR2 specifically from their intestinal epithelial cells (*Tlr2*^*ΔIEC*^) were generated by crossing a *Tlr2*-flox mouse with a Villin-Cre mouse as previously described in Ref. [38] [39] (Suppl. Fig. 1A and B). Mice globally lacking DUOX2-function due to a spontaneously occurring missense mutation at V674 (*Duox2*^*thyd/thyd*^) and C57Bl/6J mice were purchased from Jackson Laboratory, Bar Harbor, ME. USA [40]. Mice lacking DUOX2 specifically from their intestinal epithelium (*Duox2*^*ΔIEC*^) were obtained through collaboration with Felix Sommer (Institute of Experimental Medicine (IEM), Christian-Albrechts-University (CAU) Kiel, Germany) [41]. All animals were used at 8-10-weeks of age, both, male and female sex mice were used. The animals were housed in the Translational Animal Research Center of University Medical Center Mainz under specific-pathogen-free (SPF) conditions in EU type II cages with a maximum of 5 littermates receiving standard chow diet and water ad libitum at 22 °C ± 2 °C RT and a circadian 12 h light-dark cycle with 55-65% air humidity. All experimental procedures performed on mice were approved by the local committee on legislation on protection of animals (Landesuntersuchungsamt Rheinland-Pfalz G16-1-013). 3.5% dextran sodium sulfate (DSS; MP Biomedicals Germany GmbH, Eschwege, Germany) was administered orally to C57Bl/6J mice for 7 days via the drinking water to induce colitis. Successful treatment was assessed with a haemoccult test kit (Beckman Coulter Inc., Brea, CA, USA).

After euthanasia, the abdominal cavity was opened longitudinally, and the small intestine was extracted and cleaned from adipose and mesenteric tissue. Then, the lumen was flushed with ice-cold PBS (Thermo Fisher Scientific, Waltham, MA, USA) using a syringe to remove any mucus and fecal content. The small intestine was divided into 8 equal parts from proximal to distal. The mid small intestinal (Si) segment 5 was used for whole tissue analyses including histological stainings, mRNA and protein expression analyses. Primary intestinal epithelial cells (IECs) were isolated from the distal Si segments Si7-8. Here, the small intestinal segments were cut open lengthwise and incubated with 5 ml of 10 mM EDTA (AppliChem GmbH, Darmstadt, Germany) in PBS for 30 min at 37 °C on a rocking platform at 250 rpm. Samples were shaken vigorously by hand to detach the IECs from the mucosal tissue [42]. The cell suspension was centrifuged at 6500 rpm for 5 min at 4 °C to collect the IECs, which were washed with ice-cold PBS and resuspended in TRIregent (Sigma-Aldrich, St. Louis, MO, USA) or 1x RIPA lysis buffer (Merck, Darmstadt, Germany) supplemented with protease and phosphatase inhibitors (Thermo Fisher Scientific, Waltham, MA, USA) for RNA or protein extraction, respectively.

### 2.2 Cell culture

MODE-K cells were purchased from Inserm-U1111 (Dr Kaiserlian). The immortalized murine small intestinal epithelial MODE-K cell line [43] was cultured in RPMI medium supplemented with l-glutamine, sodium pyruvate, HEPES, MEM Non-essential amino acids, β-mercaptoethanol, FCS and penicillin/streptomycin (Thermo Fisher Scientific, Waltham, MA, USA) at 37 °C, 95% relative humidity and 5% CO_2_. Reaching 80% confluency, the cells were treated with 2 μg/mL of the TLR1/2 agonist Pam_3_CSK_4_ (Sigma-Aldrich, St. Louis, MO, USA) or H_2_O as control for 2 h. Following incubation, the supernatant was discarded, and cells were resuspended in 1 ml of TRIreagent for RNA isolation.

### 2.3 Patient samples

Publicly available data from single cell RNA sequencing analysis of enterocytes from Crohn's disease patients was analyzed in regard of differentially expressed genes compared to healthy controls [44]. Human subjects were recruited in the gut-clinic of the University Medical Center Mainz during a colonoscopy consultation. The collection of patient data and the acquisition of biopsies were approved upon request by the Ethics Committee of the State Medical Association of Rhineland-Palatinate (Ref.No. 837.056.15 (9824); May 26th, 2015). The informed consent was obtained at least one day prior to the colonoscopy and was conducted by the physician responsible for the intestinal outpatient clinic. Inclusion criteria for subjects with Crohn's disease were age between 18 and 75 years, clinically and histologically confirmed Crohn's disease, and at least mild inflammatory activity in the terminal ileum of the collected samples. The degree of inflammatory activity in the relevant section of the small intestine was determined based on a histological examination carried out in parallel as part of routine procedures outside the study at the Institute of Pathology at University Medical Center Mainz. Inclusion criteria for the control group were age between 18 and 75 years, no evidence of chronic inflammatory bowel disease or other pathological changes, and no previous surgeries on the colon or terminal ileum. The indication for colonoscopy in the control group was either a preventive screening or due to sporadic adenomatous polyps. Additionally, control subjects who generally took the fewest medications were selected to minimize potential interactions of drugs with the mucosa of the gastrointestinal tract. Complete exclusion criteria, for both patients with Crohn's disease and control subjects, were current pregnancy and contraindications for a colonoscopy with tissue sampling, such as ongoing therapeutic anticoagulation.

### 2.4 Immunofluorescence staining

Tissue segments of the small intestine were fixed in 37% formaldehyde (Carl Roth, Karlsruhe, Germany) overnight then embedded in paraffin. 7 μm thick sections were cut, deparaffinized, and underwent epitope retrieval in boiling citric acid (Carl Roth, Karlsruhe, Germany). Immunostaining was performed using Platelet-Endothelial Cell Adhesion Molecule 1 (PECAM-1, D8V9E, Cell Signaling, Danvers, MA, USA) as an endothelial cell marker and Lymphatic vessel endothelial hyaluronic acid receptor 1 (LYVE-1, ALY7, Thermo Fisher Scientific, Waltham, MA, USA) for lymphatic vasculature and Hoechst (Thermo Fisher Scientific, Waltham, MA, USA) for nuclei. Primary antibodies were incubated on the sections in a 1:100 dilution overnight at 4 °C. Fluorophore-conjugated secondary antibodies (Goat anti-rabbit IgG (H + L), F(ab’)2 Fragment, AF488; Goat anti-rat IgG (H + L), AF555; Cell Signaling, Danvers, MA, USA) were incubated in a 1:500 dilution for 1h at RT. Slides were visualized using an inverted fluorescence microscope (BZ-X810 Keyence, Hangzhou, China). Histological analyses were performed using ImageJ (version 1.50i; Rasband, W. S., U.S. National Institutes of Health, Bethesda, MD, USA).

### 2.5 RNA isolation and qRT-PCR

Total RNA was isolated from whole tissue or IECs using TRIreagent as previously described [45]. After assessment of RNA quality via agarose gel electrophoresis, mRNA was reverse-transcribed into cDNA using the High-Capacity cDNA Reverse Transcription Kit (Thermo Fisher Scientific, Waltham, MA, USA). qRT-PCR was performed using iTaq Universal SYBR Green Supermix (Bio-Rad Laboratories, Hercules, CA, USA) with the primer pairs detailed in Table 1. Cylcle conditions included an initial denaturation at 95 °C, followed by 44 loops of 95 °C for 10 s and 60 °C for 25 s in a qTOWER3 Real-Time PCR cycler (Analytik Jena, Jena, Germany). Samples were run in triplicates, and relative to ribosomal protein L32 gene expression levels were calculated using the 2-ΔΔCt method.

**Table 1:** Forward and reverse sequences of primers used for mRNA expression analyses.

Target gene	Forward Sequence	Reverse Sequence
*Angpt-1*	CACATAGGGTGCAGCAACCA	CGTCGTGTTCTGGAAGAATGA
*Duox2*	GCCTGGCTTTGCTCACCA	GAGGAGGAGGCTCAGGAT
*L32*	TTAAGCGAAACTGGCGGAAAC	TTGTTGCTCCCATAACCGATG
*Pdgfb*	TGTTCCAGATCTCGCGGAAC	GCGGCCACACCAGGAAG
*Pdgfr-β*	GTGGTGAACTTCCAATGGACG	GTCTGTCACTGGCTCCACCAG
*Pecam-1*	CTGCCAGTCCGAAAATGGAAC	CTTCATCCACTGGGGCTATC
*Tie2*	GCCTCCTAAGCTAACAATCTCC	GATGGCAATCGAATCACTGAAC
*Tlr2*	GCAAACGCTGTTCTGCTCAG	AGGCGTCTCCCTCTATTGTATT
*Vwf*	CTTCTGTACGCCTCAGCTATG	GCCGTTGTAATTCCCACACAAG

### 2.6 Protein isolation and Western blot

Isolated cells were resuspended in 200 μl 1x RIPA buffer (Merck, Darmstadt, Germany) supplemented with protease and phosphatase inhibitors (Thermo Fisher Scientific, Waltham, MA, USA), followed by cell lysis in an ultrasonic bath. After incubation on ice, cell lysates were centrifuged to remove any cell debris. Protein levels in the supernatant were determined using DC Protein Assay (Bio-Rad Laboratories, Hercules, CA, USA) according to manufacturer's instructions. Samples were diluted to 3.75 mg/ml in 5x reducing Laemmli buffer, followed by heat-denaturation. For Western blotting, proteins were separated according to their size by SDS-PAGE (5% stacking gel, 8% resolving gel), with a pre-stained Page Ruler protein ladder (Thermo Fisher Scientific, Waltham, MA, USA). Proteins were transferred to a 0,45 μm PVDF membrane (Merck, Darmstadt, Germany) and blocked with 5% Nonfat diary milk and 1% BSA TBST blocking buffer for 1 h at RT. Primary antibodies DUOX2 (Clone Duox S-12, Sigma-Aldrich, St. Louis, MO, USA) and β-actin (Clone 13E5, Cell Signaling, Danvers, MA, USA) were diluted 1:1000 in 1x TBST and BSA, respectively, and incubated overnight at 4 °C on a rocking platform. After washing, secondary antibodies (Rabbit anti-mouse IgG (H + L), peroxidase conjugated, Abcam, Cambridge, UK; Goat anti-rabbit IgG (H + L), peroxidase conjugated, Vector Laboratories, Newark, CA, USA) were diluted 1:10000 in 1x TBST and incubated for 90 min at RT, rocking. Protein bands were detected with Clarity Western ECL Substrate (Bio-Rad Laboratories, Hercules, CA, USA) and visualized using a Fusion FX documentation system (Vilber Lourmat, Eberhardzell, Germany). Densitometric analysis was performed using the FusionCapt Advance FX7 (v17.01, (Vilber Lourmat, Eberhardzell, Germany). All values were normalized to the respective control groups.

### 2.7 Microbiome analysis using 16S rRNA gene amplicon sequencing

Fecal content from the lumen of the mid small intestine was harvested alongside other tissues and cells from the respective mice. DNA was isolated from luminal small intestinal fecal samples using the DNeasy PowerSoil Kit (Qiagen) following the manufacturer's protocol. Extracted DNA was eluted from the spin filter silica membrane with 100 μl of elution buffer. DNA quantity and purity were assessed prior to downstream analysis. 16S rRNA gene amplicon profiling and MiSeq sequencing was performed as described earlier [46] [47] with the following modifications: the V3–V4 region of the 16S rRNA gene was amplified using the dual barcoded primers 319F (ACT CCT ACG GGA GGC AGC AG) and 806R (GGA CTA CHV GGG TWT CTA AT) [48]. Each primer contained additional sequences for a 12 base Golay barcode, Illumina adaptor, and linker sequence [49], allowing accurate multiplexing and sample identification. PCR was performed using the Phusion Hot Start Flex 2X Master Mix (NEB) in a GeneAmp PCR system 9700 (Applied Biosystems) and the following program (98 °C for 3 min, 25-30x [98 °C for 20 s, 55 °C for 30 s, 72 °C for 45 s], 72 °C for 10 min, hold at 4 °C). Performance of the PCR reactions was checked using agarose gel electrophoresis. Normalization was performed using the SequalPrep Normalization Plate Kit (Thermo Fisher Scientific, Darmstadt, Germany) following the manufacturer's instructions. Equal volumes of SequalPrep-normalized amplicons were pooled and sequenced on an Illumina MiSeq platform (2 x 300 nt). MiSeq sequence data was first subjected to quality control and sample mapping using MacQIIME v1.9.1 (http://www.wernerlab.org/software/macqiime). Briefly, sequencing reads were quality-filtered by trimming, keeping only nucleotides with a Phred quality score of at least 20, then paired-end assembled and sequences were assigned to individual samples based on barcode information. Chimeric sequences and low-quality reads were removed prior to downstream analyses. The sample-mapped MiSeq 16S rRNA gene amplicon sequence data were clustered into operational taxonomic units (OTUs) using uclust and the greengenes reference database (gg_13_8 release) with 97% identity. Representative OTUs were selected and taxonomically classified from phylum to genus level using the same database. The reference phylogenetic tree was constructed using FastTree 2. Relative taxonomic abundance was calculated by dividing OTU reads counts by the total number of sequences per sample. Microbial community structure was further characterized by beta diversity analysis using Bray Curtis dissimilarity matrix and visualized by principal coordinate analysis (PCoA). Alpha diversity metrics were also assessed to evaluate within-sample microbial richness and diversity. Differentially abundant taxa were identified using nonparametric t-tests with false discovery rate (FDR) correction for multiple comparisons. Linear discriminant analysis (LDA) effect size (LEfSe) [50] was performed using the online Galaxy platform (http://huttenhower.sph.harvard.edu/galaxy) to identify microbial taxa significantly contributing to differences between experimental genotypes. Together, these analyses provided comprehensive taxonomic characterization and comparative profiling of the intestinal microbiota composition.

### 2.8 Statistical analysis

Biostatistical analysis was performed using GraphPad Prism software (Version 10.4.2 (633) GraphPad, Inc., La Jolla, CA, USA). Comparison of two groups was done by an unpaired Student's t-test with ∗ p < 0.05, ∗∗p < 0.01, ∗∗∗p < 0.001 and ∗∗∗∗p < 0.0001. Data are presented as mean ± SD.

## 3 Results

### 3.1 Gut epithelial TLR2 impacts villus capillarization and epithelial DUOX2 expression

**Fig. 1:**
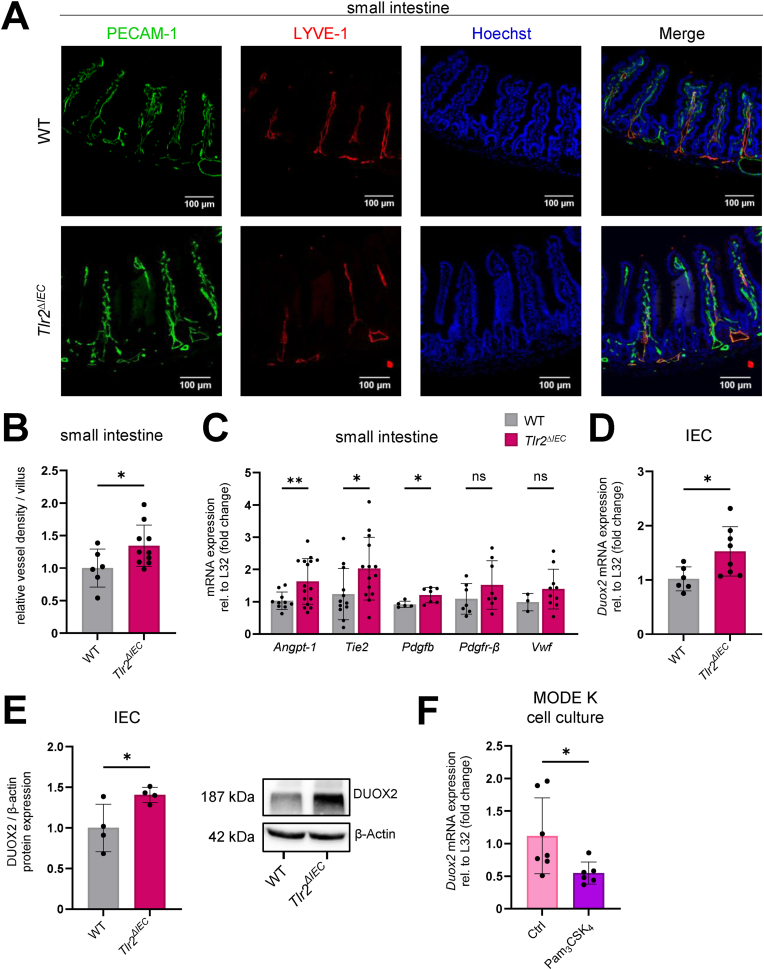
Gut epithelial TLR2 impacts villus capillarization and *Duox2* expression **A** Representative image of histological stainings showing blood capillary endothelial cells (PECAM-1, green), lacteals (LYVE-1, red) and nuclei (Hoechst, blue) in the mid small intestine of *Tlr2*^*ΔIEC*^ (pink) and WT (grey) mice, 20x magnification. **B** Quantification of PECAM-1 expression in percentage-area per villus, normalized to WT (p = 0.0488). **C** q-RT PCR analysis for angiogenic markers (*Angpt-1*, p = 0.0178; *Tie2,* p = 0.0340; *Pdgfb,* p = 0.0310; *Pdgfr-β,* p = 0.2138; *Vwf,* p = 0.3017) in the Si5 segment. **D***Duox2* mRNA (p = 0,0281), **E** DUOX2 protein (p = 0.0387) expression analysis in IECs isolated from the distal small intestine of *Tlr2*^*ΔIEC*^ and WT mice. **F***Duox2* mRNA expression analysis after Pam_3_CSK_4_ stimulation of cultured MODE K cells (p = 0.0420). Comparisons of two groups were performed via student's *t*-test. Data is presented as mean ± SD. Each data point represents a biological replicate. ∗P < 0.05; ∗∗P < 0.01; ns, no significance P > 0.05.

To pinpoint the impact of small intestinal epithelial TLR2 signaling on mucosal structures, a *Tlr2*^*ΔIEC*^ mouse line was created, lacking TLR2 expression specifically in the intestinal epithelial cells (Suppl. Fig. 1A and B). Immunofluorescence staining of the mid small intestine for the vascular marker PECAM-1 revealed that the lack of intestinal epithelial TLR2 led to a significant increase in villus capillary networks (Fig. 1A and B), whereas the expression of the lymphatic endothelial marker LYVE-1 was unchanged (Suppl. Fig. 1C). In line with increased vascularization in the small intestinal tissue of *Tlr2*^*ΔIEC*^ mice, the expression of multiple angiogenic factors was upregulated. Among those, angiopoietin 1 (*Angpt-1*) and tyrosine kinase with immunoglobulin and epidermal growth factor homology domains 2 (*Tie2)*, previously shown to be involved in microbiota-induced gut mucosal angiogenesis [20], but also platelet derived growth factor subunit B (*Pdgfb*), platelet derived growth factor receptor beta (*Pdgfr-β*), and von Willebrand factor (*Vwf*), commonly involved in vessel formation and stabilization, showed an elevating trend (Fig. 1C). Altogether, these results demonstrate an increased villus capillarization of the small intestine in *Tlr2*^*ΔIEC*^ mice.

Interestingly, the increased expression of markers was paralleled by enhanced *Duox2* expression in the small intestinal epithelium of *Tlr2*^*ΔIEC*^ mice, both on the mRNA and protein level (Fig. 1D and E). This indicates that the absence of intestinal epithelial TLR2 is linked to an enhanced *Duox2* expression. In line with these findings, the stimulation of MODE-K cells with the TLR2/1 agonist Pam_3_CSK_4_ provoked a downregulation of *Duox2*, underlining the inhibitory effect of TLR2 signaling on *Duox2* expression in small intestinal enterocytes (Fig. 1F). Taken together, these results suggest that TLR2 signaling in small intestinal epithelial cells controls gut epithelial expression of the NADPH oxidase DUOX2 and villus vascularization.

### 3.2 Intestinal epithelial *Duox2*-deficiency yields reduced small intestinal villus capillarization

**Fig. 2:**
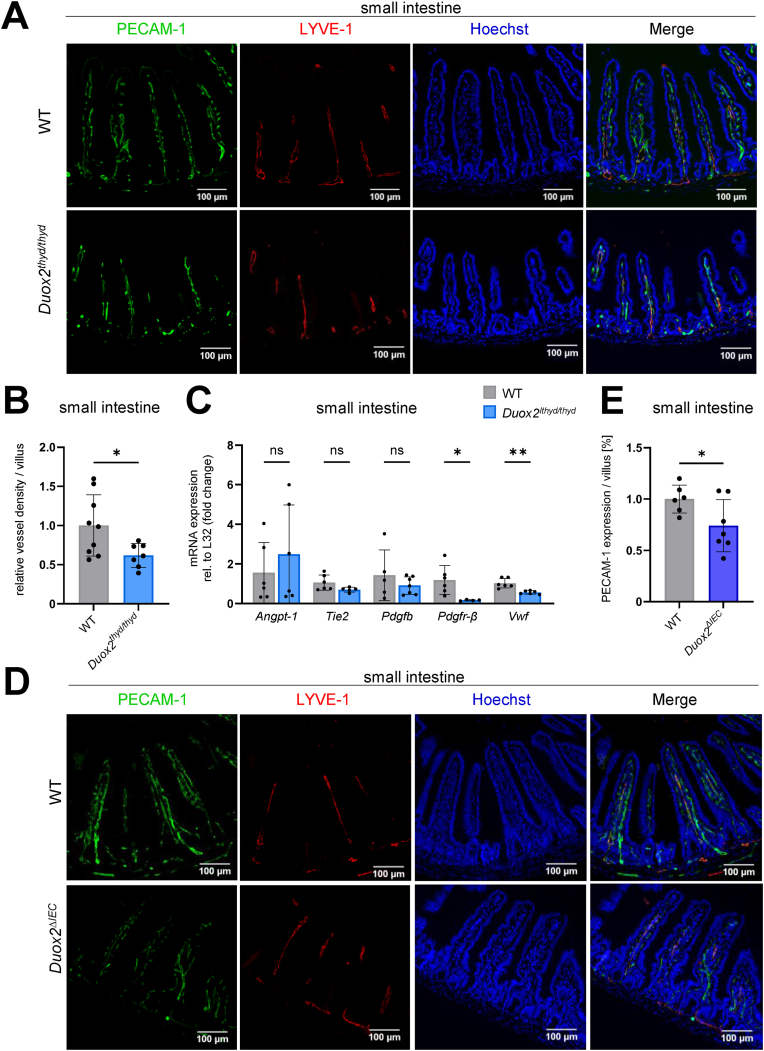
Intestinal epithelial *Duox2*-deficiency yields in reduced small intestinal villus capillarization **A** Representative image of histological stainings showing blood capillary endothelial cells (PECAM-1, green), lacteals (LYVE-1, red) and nuclei (Hoechst, blue) in the mid small intestine of *Duox2*^*thyd/thyd*^ (light blue) and WT (grey) mice, 20x magnification. **B** Quantification of PECAM-1 expression in percentage-area per villus, normalized to WT (p = 0.0292). **C** qRT PCR analysis for angiogenic markers (*Angpt-1,* p = 0.4523; *Tie2,* p = 0.0808; *Pdgfb,* p = 0.3378; *Pdgfr-β,* p = 0.0257; *Vwf,* p = 0.0012) in the Si5 segment. **D** Representative images of histological stainings showing blood capillary endothelial cells (PECAM-1, green), lacteals (LYVE-1, red) and nuclei (Hoechst, blue) in the mid small intestine of *Duox2*^*ΔIEC*^ (dark blue) and WT (grey) mice, 20x magnification. **E** Quantification of PECAM-1 expression in percentage-area per villus, normalized to WT (p = 0.0472). Comparisons of two groups were performed via student's *t*-test. Data is presented as mean ± SD. Each data point represents a biological replicate. ∗P < 0.05; ∗∗P < 0.01; ns, no significance P > 0.05.

NADPH oxidases, and especially hydrogen peroxide (H_2_O_2_), regulate mucosal angiogenesis [51] [52]. We therefore addressed whether the observed interplay between epithelial TLR2 and DUOX2 impacts gut mucosal capillarization. Since DUOX2 expression and the extent of villus capillarization were found elevated in the small intestinal epithelium of *Tlr2*^*ΔIEC*^ mice, we next analyzed vascularization in the small intestine of mutant mice that globally lack DUOX2 function (*Duox2*^*thyd/thyd*^) [40]. Although *Duox2*^*thyd/thyd*^ mice exhibit a global phenotype including hypothyroidism, dwarfism, and hearing deficiency caused by the lack of DUOX2 function during crucial steps of the thyroid hormone synthesis [40], the length of the small intestine appeared to be unaffected by these hormonal effects (Suppl. Figs. 2A, B, D-F). In line with the enhanced small intestinal *Duox2* expression and villus vascularization observed in *Tlr2*^*ΔIEC*^ mice, villus vascularization was significantly decreased upon the lack of DUOX2 function (Fig. 2A and B), while the expression of the lymphatic vascular marker LYVE-1 was not significantly altered (Suppl. Fig. 2C). This functional defect of DUOX2 was paralleled by significantly reduced expression levels of the angiogenic factors *Pdgfr-β* and *Vwf* in the mid small intestine (Fig. 2C).

Next, we controlled whether the observed vascular phenotype specifically depends on *Duox2* expression in the gut epithelial lining, thus excluding the global hormonal effects on villus vascularization. Therefore, we analyzed mice lacking *Duox2* expression specifically in the intestinal epithelium (*Duox2*^*ΔIEC*^) (Suppl. Fig. 3) [41]. In accordance with the reduced villus vascularization found in *Duox2*^*thyd/thyd*^ mice globally lacking DUOX2 function, the expression of the vascular marker PECAM-1 in the mid small intestine was significantly decreased in *Duox2*^*ΔIEC*^ mice (Fig. 2D and E). Hence, our findings highlight the critical role of the intestinal epithelial NADPH oxidase DUOX2 in villus capillarization mediated by an epithelial-to-endothelial crosstalk.

### 3.3 Defective DUOX2 function impacts composition of the gut microbiota

**Fig. 3:**
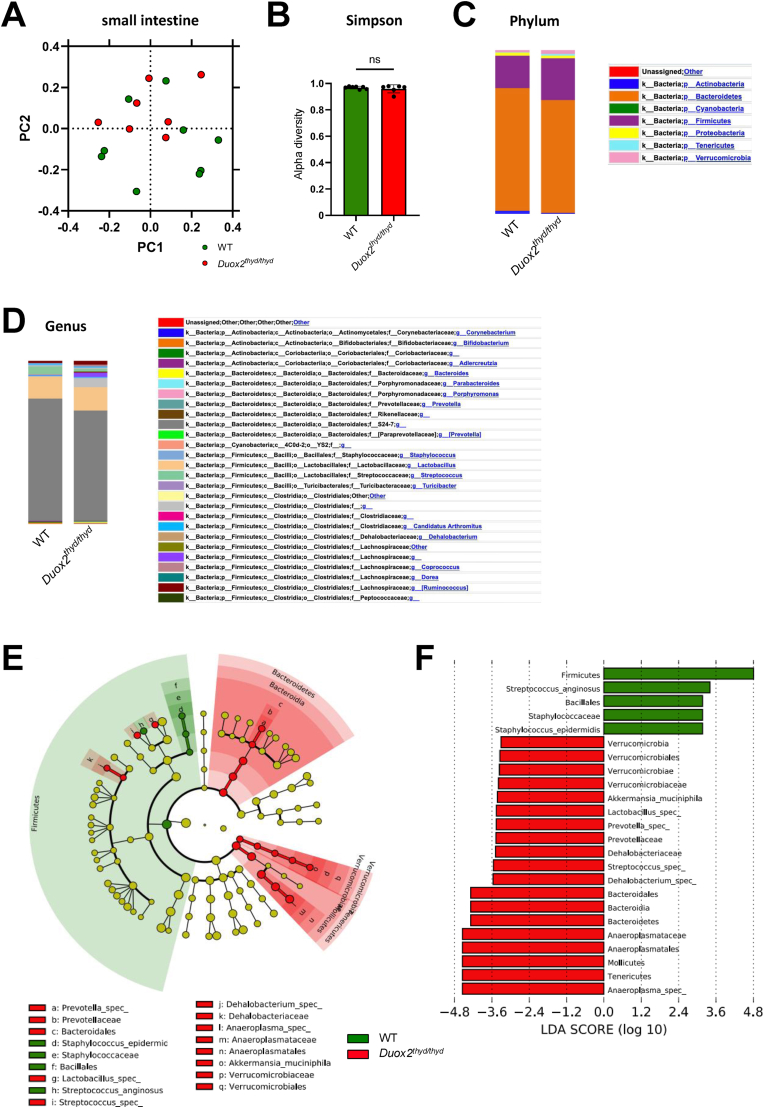
Small intestinal microbial alterations upon global DUOX2-deficiency **A** Principal coordinate analysis of the small intestinal luminal microbiome of WT (n = 7) and *Duox2*^*thyd/thyd*^ mice (n = 6) based on 16S rDNA gene profiling suggesting a genotype-effect (PERMANOVA p = 0.1489). **B** Alpha diversity using the Simpson metric (p = 0,4553). Taxonomic overview on **C** phylum and **D** on genus level. Linear discriminant analysis (LDA) effect size (Lefse) of the small intestinal microbiome of WT and *Duox2*^*thyd/thyd*^ mice highlighting bacterial taxa differences with **E** the cladogram showing the phylogenetic distribution of differential taxa and **F** differential taxa ranked by LDA score.

To investigate whether *Duox2*-deficiency affects intestinal microbial composition, comprehensive 16S rRNA gene amplicon sequencing of small intestinal luminal samples was performed, and differential taxonomic abundance was analyzed between *Duox2*^*thyd/thyd*^ and WT mice enabling taxonomic profiling at phylum, genus, and species-associated levels (Fig. 3). Unconstrained global principal coordinate analysis (PCoA) revealed a trend toward genotype-associated clustering in overall microbial community structure between WT and *Duox2*^*thyd/thyd*^ mice (Fig. 3A), although alpha diversity remained unchanged as assessed by Simpson diversity index (Fig. 3B). In contrast, relative abundance analyses demonstrated distinct genotype-dependent shifts in microbial composition at both phylum and genus levels (Fig. 3C and D). Linear discriminant analysis (LDA) effect size (LEfSe) analysis highlighted specific bacterial taxa contributing to these genotype-dependent differences (Fig. 3E and F). Loss of DUOX2 in *Duox2*^*thyd/thyd*^ mice was associated with reduction in the phylum Firmicutes, driven by *Staphylococcaceae*, and increased representation of Bacteroidetes, Tenericutes and Verrucomicrobiae driven mainly by Prevotella, *Anaeroplasma*, and *Akkermansia muciniphila*, respectively (Fig. 3E). In summary, these compositional changes suggest that DUOX2 contributes to the maintenance of small intestinal microbial homeostasis and that its loss may influence host-microbiota interactions.

### 3.4 DUOX2 is linked to villus capillarization in acute intestinal inflammation

**Fig. 4:**
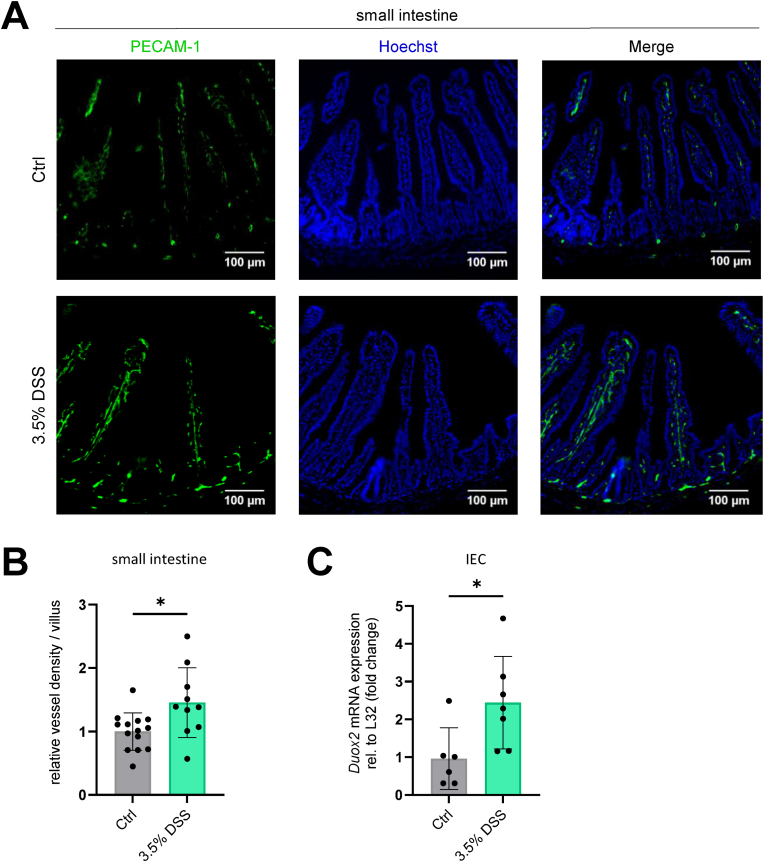
DUOX2 is linked to villus capillarization in acute intestinal inflammation 3.5% DSS treatment via drinking water of C57Bl/6J mice for 7 days to induce experimental IBD: **A** Representative images of histological stainings showing blood capillary endothelial cells (PECAM-1, green) and nuclei (Hoechst, blue) in the mid small intestine of DSS-treated and control mice, 20x magnification. **B** Quantification of PECAM-1 expression in percentage-area per villus of DSS-treated C57Bl/6J (turquoise) and control (grey) mice, normalized to controls (p = 0.0149). **C** Quantification of *Duox2* mRNA expression in IECs of DSS-treated C57Bl/6J mice (turquoise) vs controls (grey) (p = 0.0284). Comparisons of two groups were performed via student's *t*-test. Data is presented as mean ± SD. Each data point represents a biological replicate. ∗P < 0.05; ns, no significance P > 0.05.

It is well-established that DUOX2 activity is upregulated in the inflamed colon [29] [41]. Similarly, also in the small intestine DUOX2 expression has been shown to be significantly increased under inflammatory conditions, for example in Crohn's disease and ulcerative colitis [33]. Remarkably, in the analysis of publicly available single cell sequencing data of small intestinal enterocytes isolated from Crohn's patients [44], the NADPH oxidase *Duox2* and its maturation factor *Duoxa2* were observed to be among the highest upregulated genes (Suppl. Fig. 4A). This is coherent with our analyses of tissue biopsies from the terminal ileum of Crohn's disease patients since we found *Duox2* mRNA expression increased in patients with acute inflammation (Suppl. Fig. 4B, Suppl. Table 1). This finding is further supported by experimental data obtained from a murine model of acute intestinal inflammation. C57BL/6J mice were administered 3.5% DSS for 7 days, resulting in intestinal inflammation accompanied by the characteristic weight loss associated with DSS treatment (Suppl. Fig. 4C). Notably, *Duox2* mRNA expression was significantly increased in the small intestinal epithelium of DSS-treated mice (Fig. 4C), despite the characteristics of DSS inducing an intestinal inflammation mainly affecting the terminal colon. As expected, the observed increase of *Duox2* mRNA expression was associated with a marked increase in small intestinal villus capillarization (Fig. 4A–C). In conclusion, our data indicate that increased *Duox2* expression may at least in part be responsible for the altered mucosal angiogenesis observed in IBD.

## 4 Discussion

**Fig. 5:**
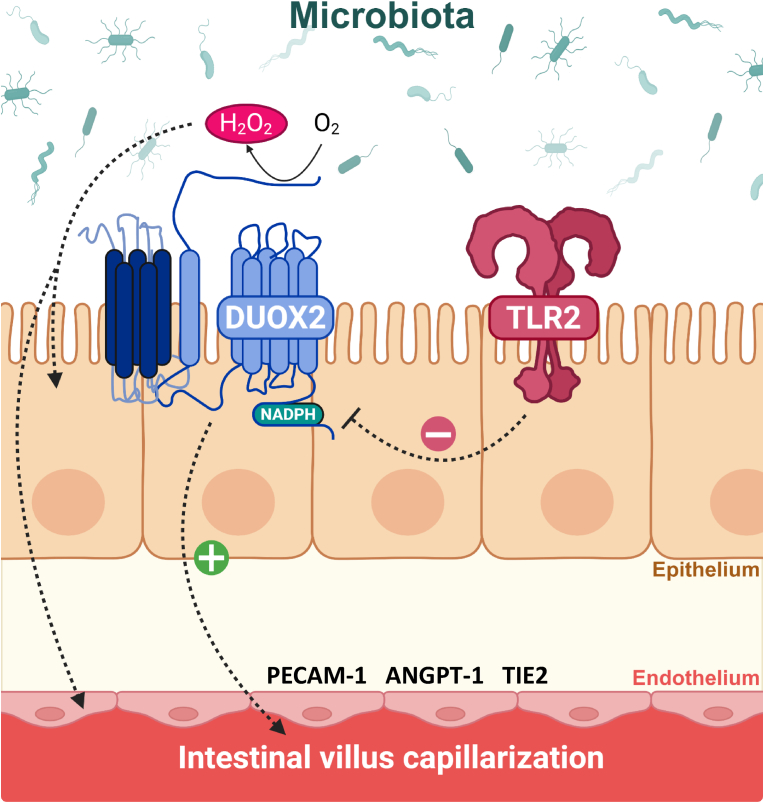
Epithelial-to-endothelial crosstalk is mediated by TLR2 and DUOX2 in the small intestine Model for gut microbiota modulating the mucosal capillarization via an epithelial-to-endothelial crosstalk mediated by expressed TLR2 and DUOX2. Gut microbial signals are transduced via TLR2 on intestinal epithelial cells affecting DUOX2 in an inhibitory manner. Alterations in DUOX2 activity in the small intestine affect the expression of angiogenic and vascular markers in underlying mucosal tissue causing remodeling of intestinal villus capillarization.

Our study delineates a novel epithelial-to-endothelial crosstalk in the small intestine linking microbial sensing via epithelial TLR2 to DUOX2-dependent redox signaling with downstream effects on small intestinal capillarization (Fig. 5). The previously unrecognized gut mucosal signaling axis constitutes a potential link between microbiota sensing by the intestinal epithelium, oxidative stress responses, and vascular remodeling, especially under inflammatory conditions.

Using gnotobiotic mouse models, the gut microbiota was repeatedly described to be a driver of small intestinal villus vascularization [19] [20] [21]. So far, the role of TLRs as regulators of gut mucosal angiogenesis remains poorly characterized [24] [53]. Of note, previous studies showed that TLR2/1 agonism induced the expression of anti-oxidative genes not only in immune cells [54], but also in the epithelium, specifically via superoxide dismutase (SOD) and glutathione peroxidase (GPx) [55] [56]. Importantly, our findings define a new role for the epithelial NADPH-oxidase in the regulation of small intestinal villus capillarization (Fig. 5) beyond the recognized DUOX2 functions in immune and oxidative pathways. The interplay between inflammation and angiogenesis in the gut is mediated through pro-inflammatory cytokines, oxidative stress, and hypoxia-inducible factors stimulating endothelial cell proliferation and neovascularization [24] [57] [58], but so far little is known on microbiota-induced pro-angiogenic mechanisms.

TLR2 expressed on the intestinal epithelium can recognize several lipoproteins, lipopeptides preferentially from gram-positive bacteria [15], having a barrier-protective role [59]. However, it is so far unclear how weakening of the gut epithelial barrier is linked to mucosal vascularization [39]. By the analysis of conditional *Tlr2*-deficient mice (*Tlr2*^*ΔIEC*^) we identified epithelial TLR2 signaling as an inhibitor of small intestinal villus capillarization. This was indicated by the expression of PECAM-1 and elevated angiogenic markers, including *Angpt-1, Tie2, Pdgfb, Pdgfr-β, and Vwf* in the small intestine of *Tlr2*^*ΔIEC*^ mice. Although TLR2/6-signaling has been associated with pro-angiogenic effects in endothelial and mesenchymal cells [58], our data suggests an indirect influence of TLR2 on submucosal vascularization through downstream regulators rather than directly signaling to the endothelium. In this context, DUOX2 emerged as a critical downstream effector. Its expression has previously been reported to be regulated by TLR-dependent MyD88/NF-κB signaling in the intestinal and colonic epithelium [35]. This hypothesis could be substantiated by the analysis of mice lacking DUOX2 function, either globally or tissue-specifically in the gut epithelium, as they showed diminished mucosal blood capillary density and decreased expression of angiogenic factors. In this context, DUOX2-derived H_2_O_2_ may serve a dual function: as a mediator of epithelial anti-microbial defense and as a signaling molecule affecting endothelial cell development and vessel formation.

ROS are important signaling mediators in the small intestine. For example, H_2_O_2_ is involved in regulating epithelial renewal, barrier function, and host-microbiota interactions [60]. In the intestinal environment, H_2_O_2_ is generated predominantly through DUOX2 activity in intestinal epithelial cells [61]. Endothelial cells, smooth muscle cells, and infiltrating immune cells, arise as additional contributors. Furthermore, mitochondria comprise an important intracellular ROS source (mtROS), especially in inflammatory conditions, where the production of mtROS was shown to have protective effects against inflammation through functional M2 macrophage polarization [62]. Beyond epithelial homeostasis, H_2_O_2_ also contributes to vascular remodeling by modulating endothelial cell proliferation, migration, and redox-sensitive signaling pathways involved in angiogenesis [63]. ROS, including H_2_O_2_, have been shown to act as second messengers in angiogenic pathways by modulating VEGF signaling and endothelial cell migration. By showing that small intestinal epithelial TLR2 signaling decreases DUOX2 expression, we provide further insight into how this pattern recognition receptor shapes the oxidative environment in the small intestinal epithelium. This provided first evidence for the functional role of gut epithelial DUOX2 in gut mucosal angiogenesis controlled by intestinal epithelial TLR2. In general, H_2_O_2_ from NADPH oxidase or mitochondrial sources has been reported to activate other sources of superoxide and hydrogen peroxide in a redox-dependent fashion, e.g., by promoting endothelial nitric oxide synthase uncoupling and conversion of the xanthine dehydrogenase to the oxidase form [64], [65]. This redox crosstalk by H_2_O_2_ comprises activation of kinases such as protein kinase C or protein tyrosine kinase 2 that regulate activity state of NOX2 or endothelial nitric oxide synthase but also proper function of mitochondrial respiratory complexes or opening of the mitochondrial permeability transition pore. Furthermore, numerous key regulators of inflammation such as NF-kB, NLRP3 or HMGB1 are redox-regulated and pro-inflammatory signaling is enhanced under oxidative stress conditions [66] [67]. Key targets of this redox-regulation are in most cases reactive cysteine residues that are either oxidized to sulfenic acid intermediates, S-glutathionylated or converted to disulfide bridges. Thereby, excessive formation of H_2_O_2_ not only controls intestinal integrity but also contributes to endothelial dysfunction, impaired mitochondrial respiration and a pro-inflammatory state.

Beyond its canonical role in host defense and maintenance of mucosal barrier integrity [13] [39] [54] [59], emerging evidence suggests that TLR2 signaling also influences epithelial differentiation, immune modulation, and redox homeostasis in the gut [68] [69] [70]. Coinciding with an increased villus vascularization, loss of epithelial TLR2 resulted in a significantly enhanced DUOX2 expression in mice. Complementary cell culture experiments showed that the stimulation of TLR2 with the TLR2/1 agonist Pam_3_CSK_4_ provoked a downregulation of *Duox2*, identifying epithelial TLR2 as an upstream regulator of DUOX2 in IECs. These reciprocal phenotypes support the hypothesis of TLR2 signaling regulating DUOX2-derived H_2_O_2_ mediating an epithelial-to-endothelial crosstalk (Fig. 5). Previous studies have shown that ROS, particularly superoxide and H_2_O_2_, can act as important signaling molecules in the microcirculation regulating vasodilation, vasoconstriction, endothelial permeability, and structural remodeling, processes that underlie angiogenesis in any vascular bed [70]. Hence, low-level H_2_O_2_ signaling, rather than high bactericidal oxidative bursts, can act as second messengers in the intestinal epithelium triggered by the gut microbiota, and mediate the epithelial-to-endothelial crosstalk.

The presence of gut microbiota does not only affect TLR2 expression in the small intestine [15], but also DUOX2 is among the strongest regulated genes upon microbial colonization in the intestinal epithelium, which underscores its importance in pathogen defense [31] [37]. DUOX2 contributes to both anti-microbial defense and H_2_O_2_ signaling. Intriguingly, the expression of *Duox2* and its maturation factor *Duoxa2* is strongly induced by the gut microbiota, both in the colon and the ileum [31] [35]. *Vice versa*, intestinal epithelial *Duox2*-deficiency impacts mucosal microbiome composition [41]. Of note, DUOX2 expression depends on MYD88 and TRIF-dependent signaling and NF-kB, a transcription factor activated through TLRs [35] [71].

Inflammation in the gut, as observed in IBD, is associated with elevated ROS levels and dysbiosis. Interestingly, epithelial TLR2 signaling is known to have a protective role against DSS-induced mucosal injury and inflammation [59]. In line with protective epithelial TLR2 signaling, our data strongly underline the contribution of intestinal epithelial DUOX2 to mucosal inflammation, which is accompanied by an increased villus vascularization. Strikingly, mice deficient in epithelial TLR2 had a higher density of capillary networks in the mid small intestinal mucosa along with elevated epithelial DUOX2 expression. Interestingly, this phenotype was also observed in the small intestine of DSS-treated mice mimicking IBD. Administration of 3.5% DSS for 7 days typically induces acute colitis characterized by progressive body weight loss, reflecting substantial intestinal inflammation and disease severity, as observed in our cohort. Furthermore, our data align with previous studies showing that DUOX2 expression is significantly increased in the intestinal epithelium of patients with Crohn's disease and in murine models of colitis induced by DSS-treatment [33] [72] [73]. Interestingly, a hallmark of Crohn's disease is increased mucosal angiogenesis, which also could be observed in the mouse small intestine in acute DSS-induced colitis [49] [74]. In these inflammatory settings, the elevated expression of *Duox2* has been associated with both microbial dysbiosis and mucosal immune activation [37] [41] [73]. Similarly, in DSS-treated mice, DUOX2 was shown to contribute to barrier dysfunction and exacerbates oxidative stress, potentially leading to chronic mucosal damage [41] [71]. Hence, DUOX2 is currently considered a strong candidate as an early microbiome-dependent functional biomarker in IBD.

Collectively, our results suggest that the epithelial TLR2–DUOX2 axis may contribute to the adaptive vascular remodeling observed in chronic intestinal inflammation, thus influencing disease progression and tissue repair. Whether this vascular response is protective or pathogenic, likely depends on the timing, localization, and intensity of DUOX2 activity. While increased vascularization may support tissue repair and immune cell recruitment, it may also sustain chronic inflammation and perpetuate chronic inflammation. Hence, our findings open several avenues for future research. Dissecting the precise molecular mechanisms and growth factor mediated signaling cues by which DUOX2 influences angiogenesis will be critical. Further, understanding how the microbiota modulates TLR2 and DUOX2 signaling in the inflamed gut could offer new insight into host–microbiome interactions that drive disease pathogenesis. Future studies should comprehensively address the involvement of mitochondrial ROS (mtROS), particularly within IECs, in the epithelial-to-endothelial signaling axis. Mitochondria-derived ROS are increasingly recognized not merely as byproducts of cell metabolism but as highly regulated signaling molecules, which can modulate transcription, inflammation, hypoxia adaptation, and intercellular communication. Lastly, therapeutic strategies targeting epithelial TLR2 signaling or modulating DUOX2 activity may provide novel approaches to ameliorate the inflammatory state while preserving mucosal healing and vascular function.

In conclusion, the intestinal epithelial TLR2–DUOX2 signaling axis emerges as a key modulator not only of innate immunity and oxidative stress but also of the vascular landscape of the gut mucosa, particularly under inflammatory conditions such as Crohn's disease. These findings underscore the integrative nature of epithelial responses to microbial cues and their impact on gut homeostasis and pathology via an epithelial-to-endothelial crosstalk.

## Funding sources

C.R. acknowledges funding from the Naturwissenschaftlich-Medizinisches Forschungszentrum (NMFZ; Johannes Gutenberg-University Mainz), the Gutenberg Research College, the Forschungsinitiative Rheinland-Pfalz and ReALity (project MORE), the BMBF Cluster4Future CurATime (project MicrobAIome; 03ZU1202CA), the DFG Research Unit INFINITE (FOR 5644), the DFG individual grant (RE 3450/17-1), and the Deutsche Zentren der Gesundheitsforschung (DZG) Innovation Fund “Microbiome” (81X2210129).

## CRediT authorship contribution statement

**Nadja Paeslack:** Data curation, Formal analysis, Investigation, Validation, Visualization, Writing – original draft. **Maximilian Mimmler:** Data curation, Formal analysis, Investigation, Validation, Visualization, Writing – original draft. **Jana Schulz:** Formal analysis, Investigation. **Julia Kownatzki:** Formal analysis, Investigation. **Bettina Kollar:** Formal analysis, Investigation, Validation. **Florentina Melzow:** Investigation. **Julie Zamor:** Investigation, Visualization. **Olga Dremova:** Formal analysis, Investigation, Methodology. **Marin Kuntic:** Investigation, Methodology. **Klytaimnistra Kiouptsi:** Investigation, Writing – review & editing. **Natalia Soshnikova:** Writing – review & editing. **Amrit Mann:** Investigation, Writing – review & editing. **Jens M. Kittner:** Investigation, Resources, Supervision, Writing – review & editing. **Andreas Daiber:** Investigation, Methodology, Supervision, Writing – review & editing. **Felix Sommer:** Investigation, Resources, Supervision, Writing – review & editing. **Christoph Reinhardt:** Conceptualization, Data curation, Formal analysis, Funding acquisition, Investigation, Methodology, Project administration, Supervision, Validation, Writing – original draft.

## Declaration of competing interest

The authors declare that they have no known competing financial interests or personal relationships that could have appeared to influence the work reported in this paper.

## Data Availability

Data will be made available on request.

## Appendix A Supplementary data

Multimedia component 1

Supplemental Figure 1 Mouse model of intestinal epithelial TLR2-deficiency A Breeding scheme for generation of *Tlr2*^flox/flox^ x Vil-Cre mice. B Quantification of *Tlr2* mRNA expression in IECs isolated from the distal small intestine of *Tlr2*^*ΔIEC*^ mice (pink) and WT littermate controls (grey) (p < 0.0001). C Quantification of LYVE-1 expression in percentage-area per villus, normalized to WT (p = 0,3816). Comparisons of two groups were performed via student's *t*-test. Data is presented as mean ± SD. Each data point represents a biological replicate. ∗P < 0.05; ∗∗P < 0.01; ∗∗∗∗P < 0.0001; ns, no significance P > 0.05. Supplemental Figure 2: Characterization of *Duox2*^*thyd/thyd*^ mice A Photographic comparison of WT (left) and *Duox2*^*thyd/thyd*^ (right) littermates. B Quantification of *Duox2* mRNA expression in Si5 harvested from *Duox2*^*thyd/thyd*^ mice (light blue) and WT littermate controls (grey) (p = 0.0443). **C** Quantification of LYVE-1 expression in percentage-area per villus, normalized to WT (p = 0.2300). Comparison of **D** bodyweights (p < 0.0001), **E** small intestinal (Si) lengths (p = 0.6688) and **F** small intestine/bodyweight (Si/BW) ratios (p < 0,0001) between *Duox2*^*thyd/thyd*^ (light blue) and WT (grey) mice. Comparisons of two groups were performed via student's *t*-test. Data is presented as mean ± SD. Each data point represents a biological replicate. ∗P < 0.05; ∗∗P < 0.01; ∗∗∗∗P < 0.0001; ns, no significance P > 0.05. Supplemental Figure 3: Characterization of *Duox2*^*ΔIEC*^ mice **A** Photographic comparison of WT (left) and *Duox2*^*ΔIEC*^ (right) littermates. **B** Quantification of *Duox2* mRNA expression in IECs isolated from the distal small intestine of *Duox2*^*ΔIEC*^ (dark blue) mice and WT littermate controls (grey) (p = 0.0018). Comparison of **C** bodyweights (p = 0.2289), **D** small intestinal (Si) lengths (p = 0.5370) and **E** small intestine/bodyweight (Si/BW) ratios (p = 0.1498) between *Duox2*^*ΔIEC*^ (dark blue) and WT (grey) mice. Comparisons of two groups were performed via student's *t*-test. Data is presented as mean ± SD. Each data point represents a biological replicate. ∗∗P < 0.01; ns, no significance P > 0.05. Supplemental Figure 4: DUOX2 is linked to villus capillarization in acute intestinal inflammation **A** Volcano plot of highest regulated genes in ileum biopsies of Crohn's disease patients (created with GEO2R from publicly available data [44]). **B** Quantification of *Duox2* mRNA expression analysis in Crohn patients (orange) terminal ileal biopsies compared to healthy controls (grey) (p = 0.0572). **C** 3.5% DSS treatment via drinking water of C57Bl/6J mice (n = 7) for 7 days to induce experimental IBD; development of bodyweight during DSS-treatment. Comparisons of two groups were performed via student's *t*-test. Data is presented as mean ± SD. Each data point represents a biological replicate. ns, no significance P > 0.05.
