# Supplementary material for: Toll-like receptor 2 impacts small intestinal villus capillarization through epithelial dual oxidase 2
Source: Redox Biol. 2026 May 14;95:104212. doi: 10.1016/j.redox.2026.104212 (PMC13262290; doi:10.1016/j.redox.2026.104212)
Supplement: Multimedia component 1 [file mmc1.docx]

| **ID** | **Sex** | **Year of Birth** | **Diagnosis** | **Remission** | **Gut Operation** | **Medication** | **Histological assessment** |
| --- | --- | --- | --- | --- | --- | --- | --- |
| C0 | f | 1986 | 2012 | no | no | 50 mg/d Solu Decortir, Buscupan, Novalgin if needed | Middle grade inflammation |
| C5 | f | 1958 | 1975 | no | no | 4mg Adalimumab/14d Bisoprolol, Pantoprazol | Low to middle grade inflammation |
| C10 | m | 1974 | 2003 | no | ileocecal resection 2008 | Adalimumab Novalgin, Tramal if needed | High grade inflammation |
| C13 | m | 1988 | 2012 | no | no | Adalimumab 40mg/ 10dOmeprazol, Alitretinoin 30mg | Middle grade inflammation |
| C15 | m | 1989 | 2010 | yes | no | Prednisolon 20mg/d, Azathiioprin 100mg/d, Pantoprazol, Novalgin if needed | Low to middle grade inflammation |
| C16 | m | 1959 | 1990 | yes | Appendectomy, ileocecal resection 2011 | Mesalazin 1,5g, Clopidogrel, Vit. B12 | High grade inflammation |
| C17 | m | 1988 | 2009 | no | appendectomy 2009, abdominal abscess 2008 | Vedalizumab/ 8 Woche | Low grade inflammation |
| C18 | m | 1946 | 1979 | yes | ileocecal resection 1995 | Adalimumab 40mg/ 14d, Ferrosanol 100mg, Carmen 10mg, Euthyrox 150mg | Low grade inflammation |
| C20 | m | 1956 | 1983 | no | Ileocecal-, sigmoid colectomy 2012 | Infliximab/ 5 weeks | Chronically active inflammation |
| K0 | m | 1939 | Control after polypectomy 2012 | X |  |  | X |
| K3 | f | 1940 |  | X |  |  | X |
| K4 | m | 1973 |  | X |  |  | X |
| K5 | f | 1956 | Prevention | X |  |  | X |
| K6 | m | 1947 | Prevention | X |  |  | X |
| K8 | m | 1971 |  | X |  |  | X |
| K10 | f | 1997 |  | X |  |  | X |
| K12 | f | 1952 |  | X |  |  | X |
| K13 | m | 1996 |  | x |  |  | x |
