# Supplementary figures and images for: Toll-like receptor 2 impacts small intestinal villus capillarization through epithelial dual oxidase 2
Source: Redox Biol. 2026 May 14;95:104212. doi: 10.1016/j.redox.2026.104212 (PMC13262290; doi:10.1016/j.redox.2026.104212)

# **SUPPLEMENT**

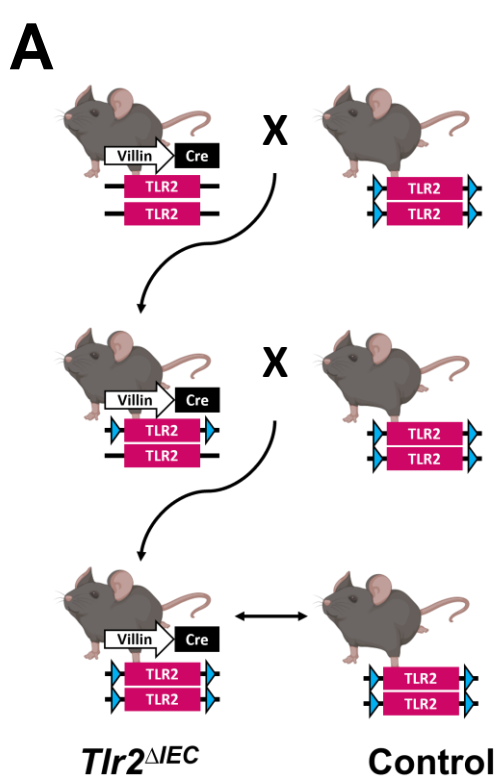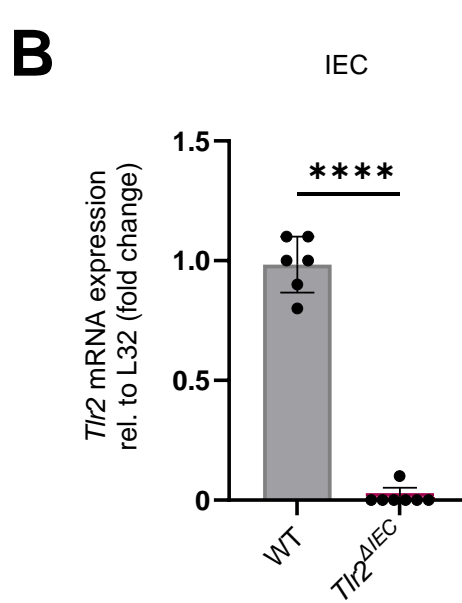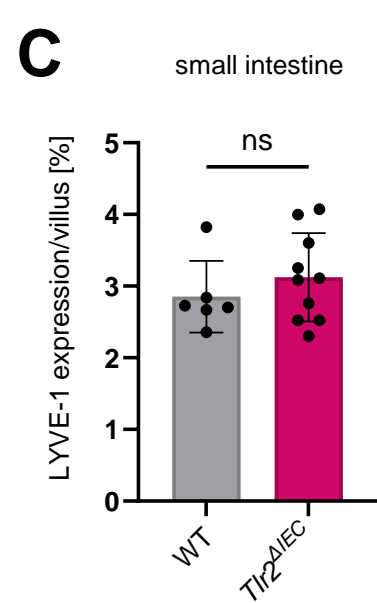

Supplemental Figure 1

**A**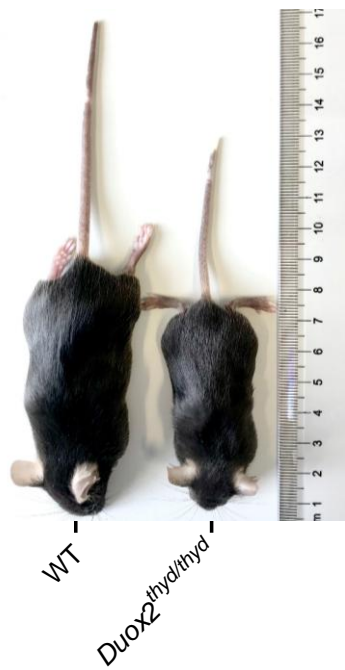**B**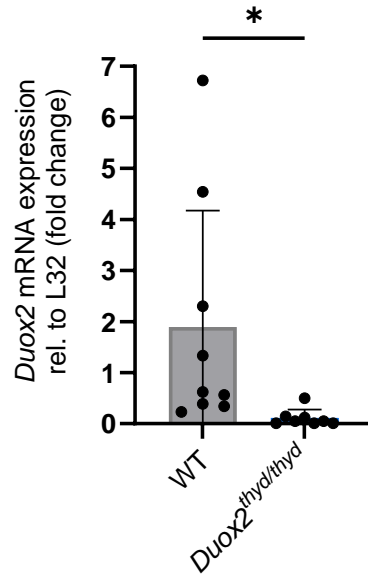**C**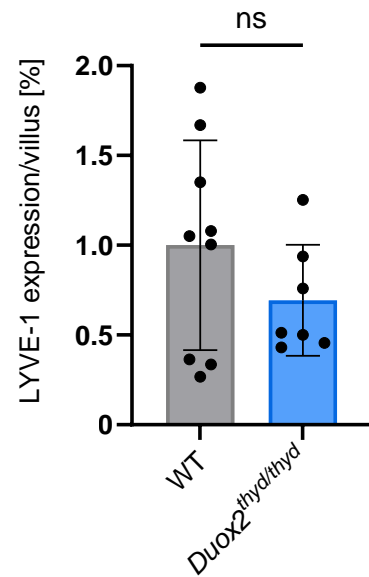**D**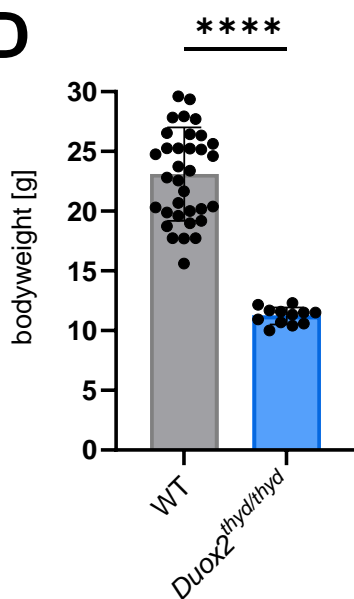**E**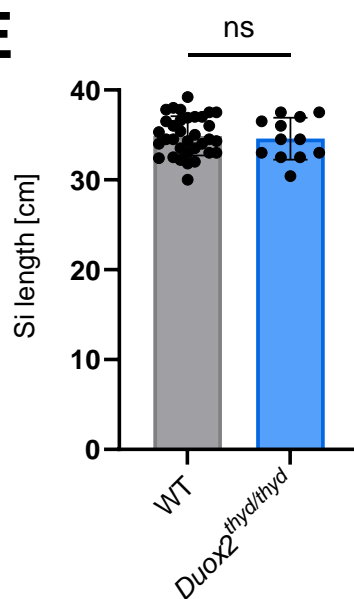**F**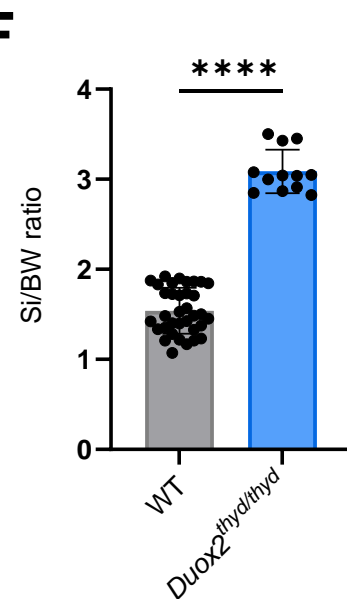

**A**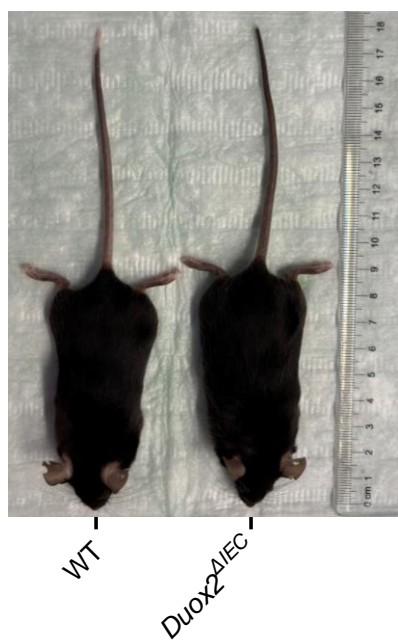**B**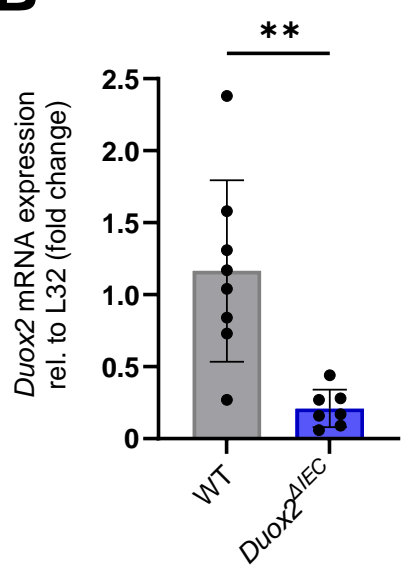**C**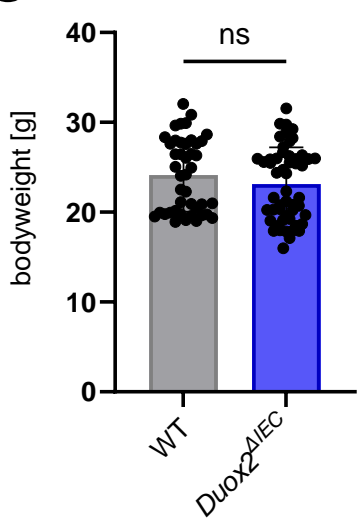**D**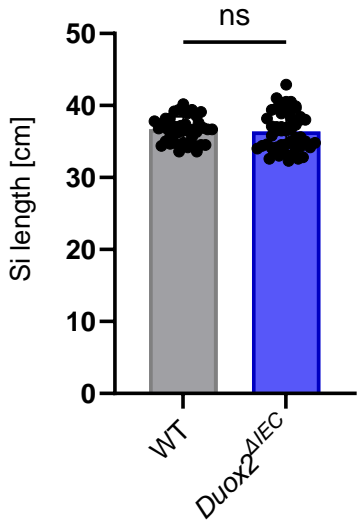**E**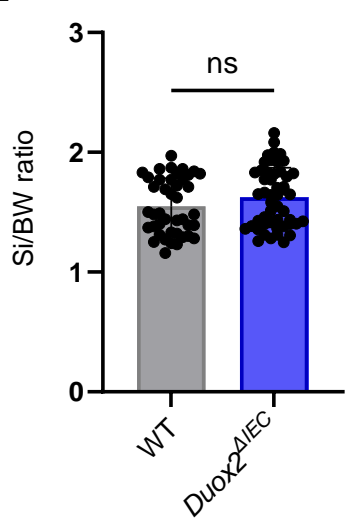

**A**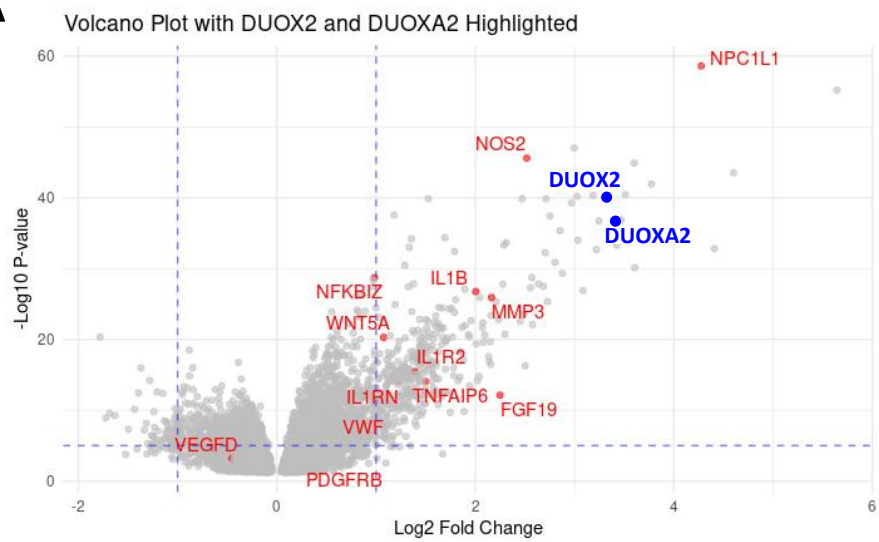**B**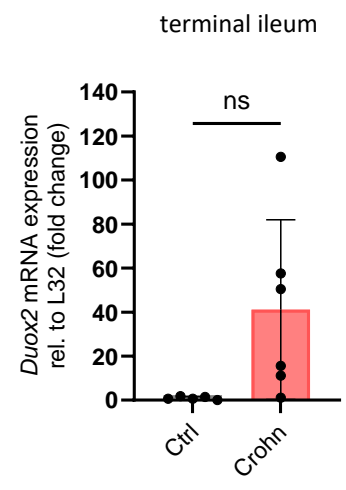**C**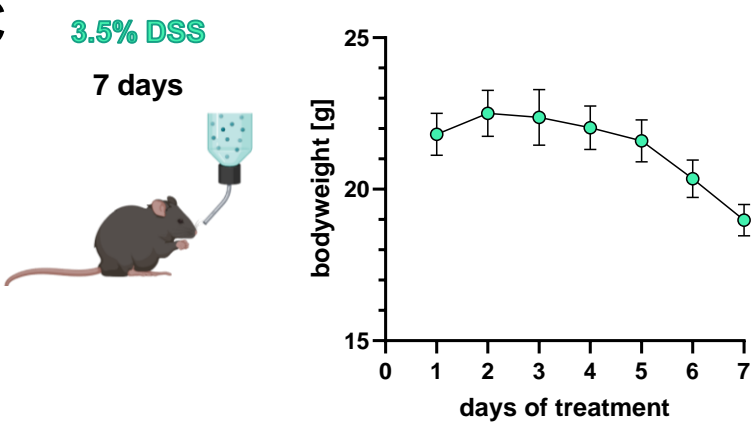

Supplement: Supplemental Figure 1 — Mouse model of intestinal epithelial TLR2-deficiency A Breeding scheme for generation of Tlr2flox/flox x Vil-Cre mice. B Quantification of Tlr2 mRNA expression in IECs isolated from the distal small intestine of Tlr2ΔIEC mice (pink) and WT littermate controls (grey) (p < 0.0001). C Quantification of LYVE-1 expression in percentage-area per villus, normalized to WT (p = 0,3816). Comparisons of two groups were performed via student's t-test. Data is presented as mean ± SD. Each data point represents a biological replicate. ∗P < 0.05; ∗∗P < 0.01; ∗∗∗∗P < 0.0001; ns, no significance P > 0.05. Supplemental Figure 2: Characterization of Duox2thyd/thyd mice A Photographic comparison of WT (left) and Duox2thyd/thyd (right) littermates. B Quantification of Duox2 mRNA expression in Si5 harvested from Duox2thyd/thyd mice (light blue) and WT littermate controls (grey) (p = 0.0443). C Quantification of LYVE-1 expression in percentage-area per villus, normalized to WT (p = 0.2300). Comparison of D bodyweights (p < 0.0001), E small intestinal (Si) lengths (p = 0.6688) and F small intestine/bodyweight (Si/BW) ratios (p < 0,0001) between Duox2thyd/thyd (light blue) and WT (grey) mice. Comparisons of two groups were performed via student's t-test. Data is presented as mean ± SD. Each data point represents a biological replicate. ∗P < 0.05; ∗∗P < 0.01; ∗∗∗∗P < 0.0001; ns, no significance P > 0.05. Supplemental Figure 3: Characterization of Duox2ΔIEC mice A Photographic comparison of WT (left) and Duox2ΔIEC (right) littermates. B Quantification of Duox2 mRNA expression in IECs isolated from the distal small intestine of Duox2ΔIEC (dark blue) mice and WT littermate controls (grey) (p = 0.0018). Comparison of C bodyweights (p = 0.2289), D small intestinal (Si) lengths (p = 0.5370) and E small intestine/bodyweight (Si/BW) ratios (p = 0.1498) between Duox2ΔIEC (dark blue) and WT (grey) mice. Comparisons of two groups were performed via student's t-test. Data is presented as mean ± SD. Each [file mmc2.pdf]
